# Salvarsan, an American Made Product

**Published:** 1917-06

**Authors:** 


### Salvarsan, an American Made Product.

It will doubtless be good news to the thousands of American physicians that Salvarsan and Neosalvarsan will henceforth be manufactured in America.

Former Congressman H. A. Metz, of New York, has just recently made the announcement that he will manufacture these products in Brooklyn, following the original Erlich process.

Dr. G. P. Metz, a well known chemist and the brother of the former Congressman, went to Germany last year and learned the process of manufacture in the laboratory where it was made so long, under the supervision of Professor Ehrlich. It will be a satisfaction to the practitioners of this country that the manufacture of this drug will no longer be guess work, but that it will be made by a highly specialized chemist who is thoroughly familiar with its manufacture.

It is now Mr. Metz's plan to give Salvarsan to hospitals and institutions as cheaply as possible and also to reduce the price as low as is consistent with the manufacture of the genuine article. The saving of a 30-cent duty, the saving of the exorbitant war risks and freight charges and the difference between the cost of manufacture abroad and at home will mean a great saving to the public.

The medical profession will soon forget that it is due to the efforts of this generous and loyal American that they have been able to get Salvarsan at so reasonable a price when everything in the drug line was soaring skyward. He steadfastly refused to raise the price just as long as possible, and then it was only a small increase. He had it in his power to have made hundreds of thousands of dollars by the sale of these two indispensable products, but he has been busying himself rather to find a way whereby he can produce them at a smaller cost.

In selling to physicians direct it is hoped to prevent speculation in these drugs, and it is deplorable that there are physicians who, while buying for their own use ostensibly, have been speculating at the expense of an ignorant public.

The manufacture of this foreign product in the United States marks a new era in the drug world. It is only just the beginning; and if the Europeans keep up the fight long enough there is no doubt that Americans will find out that after all they can get along very well without them.

Anyway, the profession as a whole appreciates the stand taken by Mr. Metz, and they will be very glad to know that Salvarsan is being manufactured by a man who has the reputation of doing thoroughly and well whatever he undertakes.

